# 

**Published:** 1940

**Authors:** 


### The Bristol Medico-Chirurgical Journal

A JOURNAL OF THE MEDICAL SCIENCES FOR THE
WEST OF ENGLAND AND SOUTH WALES.

PUBLISHED UNDER THE AUSPICES OF
*THE BRISTOL MEDICO-CHIRURGICAL SOCIETY.*

EDITOR:
J. A. NIXON, C.M.G., M.D., F.R.C.P.

WITH WHOM ARE ASSOCIATED

E. W. HEY GROVES, D.Sc., M.S., F.R.C.S.
A. E. ILES, O.B.E., M.B., B.S., F.R.C.S.
A. RENDLE SHORT, M.D., B.S., B.Sc., F.R.C.S.
W. MELVILLE CAPPER, M.B., B.S., F.R.C.S.
T. M. LING, M.D., M.R.C.P.
E. WATSON-WILLIAMS, M.C., Ch.M., F.R.C.S., Assistant Editor.

"Scire est nescire, nisi id me

Scire alius sciret."

*VOL. LVII.*

BRISTOL: J. W. ARROWSMITH LTD.

611972
Mar
24-D

WI
BR323

### Contents of Vol. LVII.

*Page*

Electro-Encephalography : An Aid to Diagnosis. By W. GREY WALTER, M.A. . . . . . . . . . . . . . . . . . . 1

Malum Immedicabile Cancer. By E. WATSON-WILLIAMS, M.C., CH.M. 9

University of Bristol Dental Hospital and School . . . . . . 41

Cerebro-Spinal Meningitis in Bristol. By B. A. PETERS, M.D., D.P.H. 77

Traumatic Extradural Hæmorrhage. Part I. By A. RENDLE SHORT, M.D., B.S., B.Sc., F.R.C.S., and MARJORIE DUNSTER, M.B., CH.B. 81

Traumatic Extradural Hæmorrhage. Part II. The clinical application of Electro-Encephalography. By W. GREY WALTER, M.A. . . 86

Head Injuries in War-time. By F. WILFRED WILLWAY, M.D., M.S., F.R.C.S. . . . . . . . . . . . . . . . . . . 91

The Twenty-ninth Long Fox Memorial Lecture : Popular Misconceptions in Medical Matters. By PROFESSOR T. B. DAVIE, M.D., F.R.C.P. . . . . . . . . . . . . . . . . . . 107

Midwifery in Bristol. By H. J. DREW SMYTHE, M.D., M.S., F.R.C.S., F.R.C.O.G., and H. L. SHEPHERD, M.B., CH.M., F.R.C.O.G. . . 117

Induction of Labour by High Puncture of the Membranes. A Survey of Eighty-seven Consecutive Cases Personally Induced during one year, November, 1938—October, 1939. By CORALIE RENDLE SHORT, M.B., CH.B., M.R.C.O.G. . . . . . . . . . . 121

THE BOMBING OF BRISTOL . . . . . . . . . . . . . . 128

THE SHELTER PROBLEM . . . . . . . . . . . . . . 130

REVIEWS OF BOOKS . . . . . . . . . . . . 46, 99, 133

EDITORIAL NOTES . . . . . . . . . . . . 54, 101, 141

OBITUARIES . . . . . . . . . . . . . . . . 58, 142

MEETINGS OF SOCIETIES . . . . . . . . . . . . 62, 143

THE MEDICAL LIBRARY OF THE UNIVERSITY OF BRISTOL 62, 102, 144

PUBLICATIONS RECEIVED . . . . . . . . . . 66, 104, 145

LOCAL MEDICAL NOTES . . . . . . . . . . 67, 105, 146

### Meetings of Societies

### Bristol Medico-Chirurgical Society.

### The Medical Library of the University of Bristol

WITH WHICH IS INCORPORATED

**The Library of the Bristol Medico-Chirurgical Society.**

*The following donations have been received since the publication of the list in December,* 1939.

April, 1940.

| | |
|---|---|
| American Laryngological Association (1) .. | 1 volume. |
| Association of American Physicians (2) .. | 1 volume. |
| The "Black Bag" (3) .. .. .. | 5 volumes. |
| British Museum (4) .. .. .. .. | 2 volumes. |
| Carnegie Institution of Washington (5) .. | 1 volume. |
| Ciba Limited (6) .. .. .. .. | 2 volumes. |
| Down Brothers Limited (7) .. .. .. | 3 volumes. |
| Miss Flemming (8) .. .. .. .. | 5 volumes. |

| | |
|---|---|
| General Medical Council (9) .. .. .. | 2 volumes. |
| Professor E. W. Hey Groves (10) .. .. | 19 volumes. |
| L'Institut Bunge (11) .. .. .. .. | 1 volume. |
| Dr. J. E. King Bequest (12) .. .. .. | 1 volume. |
| Medical Library of U.S. Army (13) .. .. | 1 volume. |
| Michell Clarke Fund (14) .. .. .. | 25 volumes. |
| Middlesex Hospital Medical School (15) .. | 2 volumes. |
| Munro Smith Fund (16) .. .. .. | 10 volumes. |
| Otago University (17) .. .. .. .. | 1 volume. |
| Dr. J. E. Shaw Bequest (18) .. .. | 12 volumes. |
| Professor A. Rendle Short (19) .. .. | 1 volume. |
| Dr. G. E. F. Sutton (20) .. .. .. | 1 volume. |
| The Vice-Chancellor (21) .. .. .. | 1 volume. |
| Stephen Zamenhof (22) .. .. .. | 1 volume. |

Unbound periodicals have also been received from Dr. R. F. Barbour, Professor E. W. Hey Groves, Professor C. Bruce Perry, Dr. G. de M. Rudolf, and Dr. J. O. Symes.

### THE ONE HUNDRED AND SEVENTIETH LIST OF BOOKS.

The figures in round brackets refer to the figures after the names of the donors. The books to which no such figures have been attached have either been bought from the Library Fund or received through the *Journal*.

| | | | |
|---|---|---|---|
| **Albee, F. H.** .. .. | *Injuries and Diseases of the Hip* .. .. | (10) | 1937 |
| **Anderson, C. G.** .. | *An Introduction to Bacteriological Chemistry* | (3) | 1938 |
| *Artificial Limbs and their relation to Amputations* .. .. .. | | (14) | 1939 |
| **Austen, E. E.** .. .. | *The House-Fly* .. .. .. .. .. | 4th Ed. (4) | 1939 |
| **Bailey, H. H.** .. .. | *Physical Signs in Clinical Surgery* .. | 7th Ed. | 1940 |
| **Bailey, H. H., and Matheson, N. M.** | *Recent Advances in Genito-Urinary Surgery* .. .. .. .. .. | (14) | 1936 |
| **Barr, J. (ed.)** .. .. | *Abrams' Methods of Diagnosis and Treatment* | (8) | 1925 |
| **Beeston, G. N.** .. .. | *Conjoint Finals* .. .. .. .. | 3rd Ed. (10) | 1938 |
| **Bourne, A. W., and Williams, L.** | *Recent Advances in Obstetrics and Gynæcology* .. .. .. .. | 4th Ed. (14) | 1939 |
| **Boyd, W.** .. .. .. .. | *Surgical Pathology* .. .. .. | 4th Ed. (14) | 1939 |
| **Brain, W. R.** .. .. | *Recent Advances in Neurology* | 4th Ed. (14) | 1940 |
| **Brauer, J. C.** .. .. | *Dentistry for Children* .. .. .. .. .. | (14) | 1939 |
| **Breen, G. E.** .. .. | *Essentials of Fevers* .. .. .. .. .. .. | (3) | 1939 |
| **Brend, W. A.** .. .. | *Handbook of Medical Jurisprudence and Toxicology* .. .. .. .. .. .. | 7th Ed. (16) | 1934 |
| **Brown, J. W.** .. .. | *Congenital Heart Disease* .. .. .. | (18) | 1939 |
| **Browne, F. J.** .. .. | *Antenatal and Postnatal Care* | 3rd Ed. (16) | 1939 |

**Burn, J. H.** .. .. *Recent Advances in Materia Medica* .. (14) 1932
**Byrne, J. G.** .. .. *Studies on the Physiology of the Eye* .. .. 1933
**Carpenter, T. M.** .. *Tables, Factors and Formulas for computing Respiratory Exchange and Biological Transformations of Energy* .. .. 3rd Ed. (5) 1939
*Ciba Hypnotics and Analgesics.* Ciba Handbook, No. 3 .. .. (6) 1939
**Clark, W. E. le Gros** .. *The Tissues of the Body* .. .. .. (16) 1939
**Cope, V. Z.** .. .. *Early Diagnosis of the Acute Adbomen* 8th Ed. 1940
**Crile, G. W.** .. .. *Hemorrhage and Transfusion* .. .. (8) 1909
**Cumberbatch, E. P.** *Essentials of Medical Electricity* 8th Ed. (14) 1939
**Dible, J. H., and Davie, T. B.** *Pathology* .. .. .. .. .. (14) 1939
**Di Natale, L.** .. .. *Il Cancero dello Stomaco* .. .. .. (10) 1939
**Down Bros.** .. .. *Catalogue of Surgical Instruments and Appliances, Aseptic Furniture, Sterilizers, etc., with appendix.* 3 Vols. .. .. (7) 1935–38
**Dunlop, D. M. and others (ed.)** *Textbook of Medical Treatment* .. (3) 1939
**Eden, T. W., and Holland, E. L.** *A Manual of Obstetrics* 8th Ed. (14) 1937
**Edwards, F. W.** .. *British Blood-Sucking Flies* .. .. .. (4) 1939
**Flagg, P. J.** .. .. *The Art of Anæsthesia* .. .. 4th Ed. (8) 1928
**Fraser, D.** .. .. .. *Clinical Studies in Epilepsy* .. .. .. (8) 1924
**Goldsmith, W. N.** .. *Recent Advances in Dermatology* .. .. (14) 1936
**Gortner, R. A.** .. .. *Outlines of Biochemistry* .. .. .. 2nd Ed. 1938
**Grimsdale H., and Brewerton, E.** *A Textbook of Ophthalmic Operations* .. .. .. .. 3rd Ed. (14) 1937
**Groves, E. W. Hey** .. *Synopsis of Surgery* .. .. 11th Ed. 1940
**Hale-White, W.** .. *Materia Medica, Pharmacy, Pharmacology and Therapeutics* .. .. .. 24th Ed. (16) 1939
**Halliburton, W. D., and McDowall, R. J. S.** *Handbook of Physiology.* 36th Ed. (16) 1939
**Hentschel, C. C., and Cook, W. R. I.** *Biology for Medical Students.* 3rd Ed. (14) 1939
**Hertzler, A. E.** .. *Surgical Operations with Local Anæsthesia* (8) 1912
**Hewer, C. L.** .. .. *Recent Advances in Anæsthesia and Analgesia* 3rd Ed. (14) 1939
**Hogben, L.** .. .. *Genetic Principles in Medicine and Social Science* .. .. .. .. .. .. .. .. 1931
**Holder, E. J.** .. .. *Surgical Sutures and Ligatures* .. .. (10) 1939
**Hutchinson, R. (ed.)** *Index of Treatment* .. .. .. 12th Ed. (10) 1940
**Illingworth, C. F. W.** *A Short Textbook of Surgery* 2nd Ed. (14) 1939
**Jamieson, E. B.** .. *Illustrations of Regional Anatomy.* 2nd Ed. Sections VI, VII .. .. .. .. .. (10) 1939
**Kerley, P.** .. .. .. *Recent Advances in Radiology* 2nd Ed. (14) 1936
**Kerr, J. M. M.** .. *Operative Obstetrics* .. .. .. 4th Ed. (14) 1937
**Kronfeld, R.** .. .. *Histopathology of the Teeth* .. 2nd Ed. (14) 1939
**Lawrence, R. D.** .. *The Diabetic Life* .. .. .. 11th Ed. (16) 1939
**McCance, R. A., and Widdowson, E. M.** *The Chemical Composition of Foods* .. .. .. .. .. .. .. .. .. 1940
**McCarrison, R.** .. .. *Food, the Foundation of Health* (Sanderson-Wells Lecture) .. .. .. .. .. (15) 1939
**McGregor, A. L.** .. *Synopsis of Surgical Anatomy* 4th Ed. (10) 1939
**McKay, W. J. S.** .. *Appendicitis* .. .. .. .. .. .. .. (10) 1936

Mackie, J. J., and McCartney, J. E. *Handbook of Practical Bacteriology.* 5th Ed. (3) 1938
May, C. H., and Worth, C. *A Manual of Diseases of the Eye* 8th Ed. (16) 1939
Miller, H. C. (ed.) .. *Functional Nerve Disease* .. .. .. (21) 1920
Moore, N. .. .. .. *History of St. Bartholomew's Hospital.* 2 Vols 1918
*New and Non-Official Remedies* .. .. .. .. .. .. .. .. (14) 1939
Ogilvie, R. F. .. .. *Pathological Histology* .. .. .. .. (3) 1940
Ogilvie, W. H. .. .. *American Journey* .. .. .. .. .. .. (10) 1937
Orr, H. W. .. .. *A New Era in the Treatment of Osteomyelitis* (10) 1930
Parsons, J. H. .. .. *Diseases of the Eye* .. .. .. 9th Ed. (16) 1938
Piney, A. .. .. .. *Recent Advances in Hæmatology* 4th Ed. (14) 1939
Ranson, S. W. .. .. *The Anatomy of the Nervous System* 6th Ed. (14) 1939
Riddell, V. H. .. .. *Blood Transfusion* .. .. .. .. .. .. .. 1939
Rogers, J. S. .. .. *Physics for Medical Students* .. 2nd Ed. 1939
Rolleston, H. D. .. *British Encyclopædia of Medical Practice.* 12 Vols. .. .. .. .. .. .. .. (18) 1936–39
Roxburgh, A. C. .. *Common Skin Diseases* .. .. .. 5th Ed. 1939
Rudolf, G. de M. (ed.) *Psychological Aspect of Delinquency* .. .. 1939
Schlosser, R. O. .. *Complete Denture Prosthesis* .. .. .. (14) 1939
Scott, S. G. .. .. *Wide Field X-ray Treatment* .. .. (10) 1939
*Sex Hormones.* Ciba Handbook, No. 4 .. .. .. .. .. .. (6) 1939
Siddle, G. H. .. .. *Technique of Stainless Steelwork in Mechanical Dentistry* .. .. .. .. .. .. .. (14) 1939
Singer, C. .. .. .. *Greek Biology and Medicine* .. .. .. (12) 1922
Smith, A. G. (ed.) .. *Irving's Anatomy Mnemonics* .. .. 4th Ed. 1939
Tidy, N. M. .. .. *Massage and Remedial Exercises* .. 4th Ed. 1939
Tredgold, A. F.. .. *Textbook of Mental Deficiency* 6th Ed. (14) 1937
Trueta, J. .. .. .. *El Tractamento de les Fractures de Guerra* (10) 1938
Trueta, J. .. .. .. *Treatment of War Wounds and Fractures* (10) 1939
Turner, J. .. .. .. *The Macewen Outlook in Surgery* .. (10) 1939
Watson-Jones, R. .. *Fractures and other Bone and Joint Injuries* (10) 1940
Webb-Johnson, A. .. *Surgery in England in the Making* (Syme Oration, 1939) .. .. .. .. .. .. (10) 1939
Whitby, L. E. H., and Britton, C. J. C. *Disorders of the Blood.* 3rd Ed. (16) 1939
Whiting, A. J., and Sutton, G. E. F. *Aids to Medical Diagnosis.* 5th Ed. 2 copies (2, 10) 1940
Whitnall, S. E.. .. *The Study of Anatomy* .. .. 4th Ed. (19) 1939
Young, J. .. .. .. *Textbook of Gynæcology* .. .. 5th Ed. (14) 1939
Zamenhof, S. .. .. *On Present Possibilities of Increasing the Higher Functions of the Cortex* .. .. .. (22) 1940

### TRANSACTIONS, REPORTS, JOURNALS, ETC.

Annual Report of the Smithsonian Institution, 1938 .. .. .. .. 1939
Die Chirurgie. Lieferung 1—2. 2te Aufl. .. .. .. .. .. .. (10) 1939
Collected Papers, Middlesex Hospital Medical School.. .. (15) 1938–39
Dentists' Register, 1940 .. .. .. .. .. .. .. .. .. .. .. .. (9) 1940
Far Eastern Association of Tropical Medicine—Transactions 9th Congress. Vol. II .. .. .. .. .. .. .. .. .. .. .. .. .. 1935

Index-Catalogue of Library of Surgeon-General's Office, U.S. Army. 4th Series. Vol. 4 .. .. .. .. .. .. .. .. .. .. (13) 1939
Medical Register, 1940 .. .. .. .. .. .. .. .. .. .. (9) 1940
Nature. Vol. 4 .. .. .. .. .. .. .. .. .. .. .. .. .. 1871
Proceedings of the University of Otago Medical School. No. 17 (17) 1940
Transactions of the American Laryngological Association. Vol. 61 (1) 1939
Transactions of the Association of American Physicians. Vol. LIV (2) 1939
Transactions of the Ophthalmological Society of the United Kingdom. Vol. LIX, Pt. II .. .. .. .. .. .. .. .. .. .. .. 1939
Travaux de l'Institut Bunge. Vol. II .. .. .. .. .. .. .. (11) 1937

### Publications Received

From MESSRS. GEORGE G. HARRAP & CO. LTD.:

*Cancer.* By GUSTAVE ROUSSY. Translated by KENNETH ELLIOTT, M.B., B.S., M.R.C.S., L.R.C.P., and JOHN DE SWITE, M.B., B.S., M.R.C.S., L.R.C.P. Price 6s.

From H. K. LEWIS & CO. LTD.:

*Practical Food Inspection.* By C. R. A. MARTIN, M.R.San.I. Second Edition. In two volumes. Volume 1—*Meat Inspection,* price 15s. Volume 2—*Fish, Poultry and other Foods,* price 12s. 6d.

From MESSRS. E. & S. LIVINGSTONE:

*A Pocket Medical Dictionary.* Compiled by LOIS OAKES, S.R.N., D.N. Assisted by T. B. DAVIE, B.A., M.D., M.R.C.P. Fourth Edition. Price 3s. 6d.

*Textbook of Public Health.* By W. M. FRAZER, O.B.E., M.D., Ch.B., M.Sc., D.P.H., and C. O. STALLYBRASS, M.D., Ch.B., D.P.H., M.R.C.S., L.R.C.P. Tenth Edition. Price 21s.

From OXFORD UNIVERSITY PRESS:

*An Introduction to Medical Genetics.* By J. A. FRASER ROBERTS, M.A., M.B., D.Sc., F.R.S.E. Price 15s.

*The Early Diagnosis of the Acute Abdomen.* By ZACHARY COPE, B.A., M.D., M.S., F.R.C.S. Eighth Edition. Price 10s. 6d.

*British Red Cross Society Notebook, with Diagrams for use during attendance at Red Cross courses of First Aid.* Ed. by ST. J. D. BUXTON, M.B., B.S., F.R.C.S. Second Edition. Price 2s. 6d.

*Tuberculosis of Bone and Joint.* By G. R. GIRDLESTONE, M.A., B.M., F.R.C.S. Price 30s.

*The Sexual Perversions and Abnormalities.* By CLIFFORD ALLEN, M.D., M.R.C.P., D.P.M. Price 7s. 6d.

*Surgery of the Hand.* By JOHN HAROLD COUCH, M.A., M.B., F.R.C.S. Price 7s.

*A Textbook of Psychiatry for Students and Practitioners.* By D. K. HENDERSON, M.D., F.R.F.P.S., F.R.C.P.E., and R. D. GILLESPIE, M.D., F.R.C.P., D.P.M. Fifth Edition. Price 20s.

From MESSRS. JOHN WRIGHT & SONS LTD. :

*British Journal of Surgery*. No. 107. Price 12s. 6d.

*Leprosy*. By SIR LEONARD ROGERS, K.C.S.I., C.I.E., M.D., F.R.C.P., F.R.C.S., F.R.S., I.M.S., and ERNEST MUIR, C.I.E., M.D., F.R.C.S. Second Edition. Price 15s.

*The Surgery of the Alimentary Tract*. By SIR HUGH DEVINE, M.S., F.R.A.C.S., F.A.C.S. Price 63s.

### Local Medical Notes

THE twenty-ninth Long Fox Memorial Lecture will be delivered in the University on Tuesday, 2nd July, at 8.30 p.m. by Professor T. B. Davie. The subject will be announced later.

NEW ARRANGEMENTS FOR OUT-PATIENTS.

The amalgamation of the Bristol Royal Infirmary and Bristol General Hospital to form the Bristol Royal Hospital has now reached a stage at which it may be useful to give a short account of local medical arrangements.

**From 1st June Out-patients** will be seen as follows :—

| *At Royal Infirmary.* | *At General Hospital.* |
|---|---|
| Medical. | Gynæcological. |
| Surgical. | Skin Diseases. |
| Orthopædic. | Ear, Nose and Throat. |
| Fractures. | Surgical (probably). |
| (Casualties). | (Casualties). |

All Eye cases at Eye Hospital only.

All Dental cases at Dental Hospital only.

**Midwifery In-patients** will be admitted to the Maternity Hospital, Southwell Street, or to Southmead Hospital *only* : domiciliary midwifery is in the charge of the M.O.H., and ante-natal attention is given at the Central Health Clinic, Tower Hill, and the other municipal clinics.

### APPOINTMENTS.

The following appointments have been made in the University Dental Hospital and School:—

Honorary Dental Surgeons and Clinical Teachers in Dental Surgery: Messrs. G. F. Fawn, N. H. Kettlewell, F. R. Hogbin, W. J. Lennox, J. Wells, N. H. Simmonds, S. K. Rigg, L. E. Claremont, Trevor Johnson, R. H. McKeag and J. W. E. Snawdon. Dental Assistant - Dean and Lecturer in Dental Surgery: P. J. Stoy. Lecturer in Dental Prosthesis: R. B. Pickles. Senior House Surgeon: Mr. R. S. Martin; Junior House Surgeon, Mr. G. R. Styles. Almoner Secretary: Miss M. G. Cornwall Jones, M.A. Sister: Miss E. G. Thomson.

---

### EXAMINATION RESULTS.

**University of Bristol.**—Students of the University have recently passed the following examinations:—

M.B., Ch.B.—*Final Examination. Pass:* W. H. G. Elliott.

**University of Dublin.**—M.D. (*by Thesis*)—R. E. Hemphill, M.B., B.Ch., B.A.O.

**Conjoint Board.**—L.R.C.P., M.R.C.S.—*First Examination. Pass:* B. L. Finzel (Physiology).

L.R.C.P., M.R.C.S.—*Final Examination.* D. L. Bayley* (Medicine).

L.D.S., R.C.S.—*Final Examination. Pass Part I:* G. R. Styles. *Part II:* G. R. Styles,* S. G. Sheffield.*

**Society of Apothecaries, London.**—L.M.S.S.A.—*Final Examination. Pass:* E. A. Griffiths* (Midwifery).

* Qualified.

### Bristol Medico-Chirurgical Society.

### President.

L. A. MOORE, M.B., Ch.B. Brist.

### President-Elect.
C. CORFIELD, M.D. Durh.

### Committee.

*Ex-Officio* { J. A. NIXON, *C.M.G.*, B.A., M.D. Cantab., F.R.C.P. (Editor of *Journal*).

(Vacant), (Hon. Librarian).

*Elected.*

C. A. MOORE, M.B., M.S. Lond., (Ex-President).
R. C. CLARKE, *O.B.E.*, M.B., Ch.B.Brist.
H. H. CARLETON, M.A., M.D., B.Ch.Oxon.
C. CORFIELD, M.D. Durh.
A. L. TAYLOR, M.D. Lond.
P. S. MARTIN, *M.C.*
T. MILLING, M.B., Ch.B. Belf.
W. V. WOOD, *M.C.*, B.A. Cantab.
H. L. SHEPHERD, M.B., Ch.M. Brist.
W. A. JACKMAN, F.R.C.S.

### Honorary Secretary.

R. V. COOKE, Ch.M.

### Honorary Treasurer.

A. E. ILES, *O.B.E.*, M.B., B.S. Lond., F.R.C.S.

### University Library Sub-Committee.
A. R. SHORT, B.Sc., M.D. Lond., F.R.C.S.
C. BRUCE PERRY, M.D. Brist., F.R.C.P.
J. H. DREW SMYTHE, *M.C.*, M.S., M.D. Lond., F.R.C.S., F.C.O.G.

Medical Practitioners wishing to join the Society are requested to communicate with the Hon. Secretary, Mr. R. V. Cooke, Failand Lodge, Guthrie Road, Clifton. The Annual Subscription is £1 1s., payable to Hon. Treasurer.

---

*Extracts from Journal By-laws.*

4.—The *Journal* shall be delivered free to Subscribers and also to Members of the Society whose subscriptions are not in arrear.

6.—The Annual Subscription to the *Journal* is 10/6, payable to Hon. Treasurer.

---

Communications intended for insertion in the *Journal* should be sent to the Editor, Dr. J. A. Nixon, 7 Lansdown Place, Clifton, Bristol.

Books for Review and Exchange Journals should be sent to the Assistant Editor, *Bristol Medico-Chirurgical Journal*, Medical Library, The University, Bristol. Reviews should be returned with the books to Dr. T. M. Ling, 50 Pembroke Road, Bristol 8.

Communications referring to the delivery of the *Journal* should be sent to the Hon. Treasurer, Mr. A. E. Iles, 87 Pembroke Road, Bristol 8.

Cases for Binding Vols. I—LVI are now ready, and may be obtained price 2/6 each (postage extra), upon application to J. W. Arrowsmith Ltd., Quay Street, Bristol; or the *Journal* will be bound, including Case, for 7/- per volume. The inclusive index for Volumes I—L may be obtained, price 3/- (postage extra).

### Bristol Medico-Chirurgical Society.

### PAST PRESIDENTS.

1874—75. FREDERICK BRITTAN, M.D., B.S. Dub.
1875—76. ROBERT WILLIAM COE.
1876—77. SAMUEL MARTYN, M.D. Ed.
1877—78. AUGUSTIN PRICHARD, M.D. Berlin.
1878—79. HENRY EDWARD FRIPP, M.D. Würzburg.
1879—80. WILLIAM MICHELL CLARKE.
1880—81. JOSEPH GRIFFITHS SWAYNE, M.D. Lond.
1881—82. EDWARD LONG FOX, M.D. Oxon.
1882—83. JAMES GEORGE DAVEY, M.D. St. And.
1883—84. WILLIAM JOHNSTONE FYFFE, M.D., B.A. Dub.
1884—85. GEORGE FORSTER BURDER, M.D. Aberd.
1885—86. WILLIAM HENRY SPENCER, M.D., M.A. Cantab.
1886—87. FRANCIS POOLE LANSDOWN.
1887—88. LEMUEL MATTHEWS GRIFFITHS.
1888—89. ROBERT SHINGLETON SMITH, M.D., B.Sc. Lond.
1889—90. NELSON CONGREVE DOBSON, Ch.M. Brist.
1890—91. SAMUEL HENRY SWAYNE.
1891—92. FRANCIS RICHARDSON CROSS, M.B. Lond., LL.D. Brist.
1892—93. EDWARD MARKHAM SKERRITT, M.D., B.S., B.A. Lond.
1893—94. JAMES GREIG SMITH, M.B., C.M., M.A. Aberd., F.R.S.E.
1894—95. ALFRED JAMES HARRISON, M.B. Lond.
1895—96. ARTHUR WILLIAM PRICHARD.
1896—97. ALFRED EDWARD AUST LAWRENCE, M.D., C.M. Aberd.
1897—98. JOHN EDWARD SHAW, M.B., C.M. Ed.
1898—99. ROBERT ROXBURGH, M.B. Ed.
1899—00. WILLIAM HENRY HARSANT.
1900—01. DAVID SAMUEL DAVIES, M.D. Lond.
1901—02. BARCLAY JOSIAH BARON, M.B., C.M. Ed.
1902—03. GEORGE MUNRO SMITH, M.D. Brist.
1903—04. JAMES PAUL BUSH, C.M.G., C.B.E., Ch.M. Brist
1904—05. REGINALD EAGER, M.D. Lond.
1905—06. JOHN DACRE.
1906—07. JAMES TAYLOR.
1907—08. HENRY WALDO, M.D., C.M. Aberd.
1908—09. JOHN MICHELL CLARKE, M.D., M.A. Cantab.
1909—10. JAMES SWAIN, C.B., C.B.E., M.D., M.S. Lond.
1910—11. BERTRAM MITFORD HERON ROGERS, M.D., B.Ch., B.A. Oxon.
1911—12. CHARLES ALEXANDER MORTON.
1912—13. WALTER CARLESS SWAYNE, M.D., B.S. Lond. and Brist.
1913—14. PATRICK WATSON-WILLIAMS, M.D. Lond., M.D. (Hon. Causa) Brist.
1914—15. WILLIAM HARRY CHRISTOPHER NEWNHAM, M.A., M.B. Cantab.
1915—16. GEORGE PARKER, M.A., M.D. Cantab., LL.D. Brist.
1916—17. FRANCIS HENRY EDGEWORTH, M.A., M.D. Cantab., D.Sc. Lond.
1917—18. FREDERICK LACE.
1918—19. ROBERT GUTHRIE POOLE LANSDOWN, M.D., B.S. Durh.
1919—20. I. WALKER HALL, M.D., Ch.B. Vict.
1920—21. LEOPOLD ERNEST VALENTINE EVERY-CLAYTON, M.D. Lond.
1921—22. CYRIL HUTCHINSON WALKER, B.A., M.B. Cantab.
1922—23. JOHN ALEXANDER NIXON, C.M.G., B.A., M.D. Cantab.
1923—24. JOHN LACY FIRTH, M.D., M.S. Lond.
1924—25. JOHN ODERY SYMES, M.D. Lond.
1925—26. THOMAS CARWARDINE, M.S. Lond.
1926—27. ARTHUR LAUNCELOT FLEMMING, M.B., Ch.B. Brist.
1927—28. WALTER KENNETH WILLS, M.A., M.B., B.Ch. Cantab.
1928—29. HENRY LAWRENCE ORMEROD, M.D., B.Ch. R.U.I.
1929—30. CHARLES FERRIER WALTERS.
1930—31. DAVID CHARLES RAYNER, Ch.M. Brist.
1931—32. JOHN ROGER CHARLES, M.A., M.D. Cantab.
1932—33. ERNEST WILLIAM HEY GROVES, M.D., M.S., D.Sc., B.Sc. Lond.
1933—34. HERBERT ELWIN HARRIS, B.A., M.B. Cantab.
1934—35. A. RENDLE SHORT, B.Sc., M.D. Lond.
1935—36. ARTHUR ERNEST ILES, O.B.E., M.B., B.S. Lond.
1936—37. RICHARD CHRISTOPHER CLARKE, O.B.E, M.B. Lond.
1937—38. CLIFFORD A. MOORE, M.B., M.S. Lond.
1938—39. LEONARD AUGUSTUS MOORE, M.B., Ch.B. Brist.

### 1940.

### LIST OF SUBSCRIBERS

TO THE

### Bristol Medico-Chirurgical Journal.

### HONORARY MEMBER OF THE SOCIETY.

Swain, James, *C.B.*, *C.B.E.*, M.D., M.S. Lond. .. .. .. Wyndham House, Easton-in-Gordano.

### SUBSCRIBERS.

*Those marked * are Members of the Society.*

| | |
|---|---|
| *Adams, A. W., M.S. Lond. .. .. .. .. | Rodney House, Clifton, Bristol 8. |
| Adams, J. B. .. .. .. .. .. .. .. .. | Kent Lodge, Eastbourne. |
| *Adams, S.B., Ph.D., M.B., Ch.B. Brist. .. .. | 19 Charlotte Street, Bristol 1. |
| *Aldwinckle, Helen M., M.B., Ch.B. Brist. .. | 23 Rockland Road, Downend, Bristol. |
| *Alexander, A. J. P., M.D. Belf. .. .. .. | Winsley Sanatorium, near Bath. |
| *Alexander, D. A., M.B., Ch.B. Vict. .. .. | 112 Pembroke Road, Clifton, Bristol 8. |
| Alexander, G. L., B.A., M.B., B.Ch. Cantab. | West African Medical Service, Laura, via Accra, Gold Coast. |
| *Alford, A. F., M.B., Ch.B. Brist. .. .. .. | Board of Education, Kingsway, London, W.C.2. |
| Alford, H. J., M.D. Lond. .. .. .. .. | 4 Park Terrace, Taunton. |
| *Armstrong, Jean, M.B., Ch.B. Brist. .. .. | Succoth, Duckmoor Road, Ashton Gate, Bristol 3. |
| Ashton, L. P., M.B., Ch.B. Brist. .. .. .. | Kapsowar, P.O. Eldoret, Kenya, East Africa. |
| *Aubrey, T., M.B. Lond. .. .. .. .. .. | Bitton, near Bristol. |
| *Bain, W. .. .. .. .. .. .. .. .. .. | 1 Greville Road, Southville, Bristol 3. |
| *Baker, Lily A., M.D., B.Ch., B.A.O., R.U.I. | 23 Pembroke Road, Clifton, Bristol 8. |
| *Barber, M. C., M.B., Ch.B. Brist. .. .. .. | Ingledene, High Street, Staple Hill, Bristol. |
| *Barbour, R. F., M.A. Cantab., M.B., Ch.B. | 16 Mortimer Road, Clifton, Bristol. |
| *Barker, G. H., M.D., B.Sc. Lond. .. .. .. | 124 Redland Road, Bristol 6. |
| *Bates, R. M. .. .. .. .. .. .. .. .. | Purdown House, Stapleton, Bristol 5. |
| *Baxter, Ursula, M.B., Ch.B. Brist. .. .. | 167 Wells Road, Knowle, Bristol 4. |
| *Baxter, V. T., M.B., Ch.B. .. .. .. .. | 167 Wells Road, Knowle, Bristol 4. |
| *Beatson, D. H., M.B., Ch.B. Brist. .. .. | 8 Richmond Park Road, Clifton, Bristol 8. |
| *Bell, R. J. Irving .. .. .. .. .. .. .. | 5a Oakfield Road, Clifton, Bristol 8. |
| *Bergin, F. G. .. .. .. .. .. .. .. .. | 31 Oakfield Road, Clifton, Bristol 8. |
| *Berry, R. J. A., Prof., M.D., C.M. Edin. .. | 320 Canford Lane, Bristol 9. |
| *Birrell, J. A., M.D., B.S. Lond. .. .. .. | 1 Victoria Square, Bristol 8. |
| Blackett, J. F., M.D., B.S. Lond. .. .. .. | Hamsterley, Bloomfield Park, Bath. |
| Bletchly, G. Playne, M.B. Lond. .. .. | c/o Lloyds Bank, Nailsworth, Glos. |
| *Bodman, A. P., M.B., Ch.B. .. .. .. .. | 11 Hurle Crescent, Clifton, Bristol 8. |
| *Bodman, F. H., M.D., Ch.B. Brist. .. .. .. | 2 Redland Court Road, Bristol 6. |
| Bolt, R. F. .. .. .. .. .. .. .. .. .. | 70 Great Russell Street, London, W.C.1. |
| *Boulton, B. J., M.B., Ch.B. Brist. .. .. | Ham Green Hospital, Pill, near Bristol. |
| *Bown, Naomi J., M.B., Ch.B. Brist. .. .. | The Corner, Nailsea, Somerset. |
| *Bradbeer, E. G., M.B., Ch.B. Brist. .. .. | 13 Percival Road, Bristol 8. |
| Brasher, C. W. J. .. .. .. .. .. .. .. | 62 Queen Anne St., Cavendish Square, London, W.1. |
| *Brinckman, Winifred P., M.B., Ch.B. Brist. | 4 Oakland Road, Redland, Bristol 6. |
| *Britton, R. B., M.B., B.S. Lond. .. .. .. | 33a Whiteladies Road, Clifton, Bristol 8. |
| *Brocklehurst, R. J., M.A., D.M. Oxon. .. | The University, Bristol 8. |
| Brown, J. E., M.B., Ch.B. Brist. .. .. .. | Coulthurst Villa, Brives Road, Maraval, Trinidad, B.W.I. |
| Brown, James M. .. .. .. .. .. .. .. | Medical Director, c/o Reed & Carnrick, 155–159 Van Wagenen Ave., Jersey City, N.J., U.S.A. |
| *Browne, R. C. .. .. .. .. .. .. .. .. | Penhill, Shirehampton, Bristol. |

| | |
|---|---|
| *BURGES, R. | Druid's Mead, Stoke Bishop, Bristol 9. |
| *BURGESS, N. F. C., M.D. Cantab. | Hereford House, Clifton, Bristol 8. |
| *BUSH, G. B., M.B., Ch.B. Brist. | Litfield House, Clifton, Bristol 8. |
| *CAIRNS, F. J. | Devon House, Whitehall Road, Bristol 5. |
| *CAPPER, W. M., M.B., B.S. Lond. | Bristol Royal Infirmary, Bristol. |
| *CAREY, R. S., *O.B.E.*, B.A. Cantab. | 502 Bath Road, Brislington, Bristol 4. |
| *CARLETON, H. H., M.A., M.D., B.Ch. Oxon. | 1 Lansdown Road, Clifton, Bristol 8. |
| *CASSON, E., M.D., Ch.B. Brist. | Dorset House, Clifton Down, Bristol 8. |
| *CATES, H. J., M.D. Lond. | Northwoods House, Winterbourne, Bristol. |
| *CHAMBERS, E. R. | 9 Clifton Park, Clifton, Bristol 8. |
| *CHARLES, J. R., M.A., M.D. Cantab. | Litfield House, Clifton Down, Bristol 8. |
| *CHITTY, H., M.S. Lond. | 46 Pembroke Road, Clifton, Bristol 8. |
| *CHRISTOFFERSON, P. E., M.B., Ch.B. Brist. | 4 Victoria Square, Clifton, Bristol 8. |
| *CLAREMONT, L. E., M.D.S. Brist. | 5 Rodney Place, Clifton, Bristol 8. |
| *CLARK, DENNIS O. | 168 Milton Road, Bristol Road, Weston-super-Mare. |
| *CLARKE, R. C., *O.B.E.*, M.B., Ch.B. Brist. | 29 Victoria Square, Clifton, Bristol 8. |
| *CLUTTERBUCK, E. R., M.B., Ch.B. Brist. | 63 Walliscote Road, Weston-super-Mare. |
| *COATES, H. | Notts County Mental Hospital, Radcliffe-on-Trent. |
| *COCHRANE, J. H. | 11 Bryant's Hill, St. George, Bristol 5. |
| *COLLINGWOOD, F. C., M.B., Ch.B. Brist. | 7 Hurle Crescent, Clifton, Bristol 8. |
| CONNELLAN, P. S. | Marmion, Shortheath Road, Farnham, Surrey. |
| *COOKE, ELIZABETH M., M.D. Lond. | Failand Lodge, Guthrie Road, Clifton. |
| *COOKE, R. V., Ch.M. Brist. | Failand Lodge, Guthrie Road, Clifton. |
| *COOKSON, R. G. | 5 Worcester Road, Clifton, Bristol 8. |
| *COOMBS, C. F., B.Ch. Cantab. | 14 Southfield Road, Westbury-on-Trym, Bristol. |
| COOMBS, N. C. | Romaldkirk, Barnard Castle, Co. Durham. |
| *COOPER, W. R. | 13 Westfield Park, Redland, Bristol 6. |
| *CORFIELD, C., M.D. Durh. | 5 Kensington Place, Clifton, Bristol 8. |
| *CORNER, BERYL, M.D., B.S. Lond. | 3 Elton House, Rodney Place, Bristol 8. |
| *COSSHAM, W. L., M.B., Ch.B. Brist. | 3 Claremont Road, Bishopston, Bristol 7. |
| *COURTNEY, R. M., M.B., Ch.B. Glasg. | Manor House, Southmead Road, Bristol |
| *COXON, BEATRICE | 16 Richmond Hill, Clifton, Bristol 8. |
| *CRAIG, ANNE A., M.D., B.S. Lond. | 35 Queen's Court, Clifton, Bristol 8. |
| *CROOK, B. A., M.B., Ch.B. Brist. | The Laurels, Timsbury, near Bath. |
| *CROSSMAN, F. W., M.B. Durh. | White's Hill, Hambrook, near Bristol. |
| *DATTA, S. S., M.D. Brist. | Snowdon House, Fishponds, Bristol. |
| *DAVIES, E. D. D. | Standish House, Stonehouse, Glos. |
| *DAWSON, W. R., *O.B.E.*, M.D. Dub. | 18 Brock Street, Bath. |
| *DELANEY, N., M.B., Ch.B. Manch. | The Meade, Bishopsworth, Somerset. |
| *DELICATI, J. L. | Royal Mineral Water Hospital, Bath. |
| *DICKSON, W. A., M.D. St. And. | Standish House, Stonehouse, Glos. |
| *DISNEY, M., M.D. Lond. | Bristol Royal Infirmary. |
| *DIX, C. | 14 Mortimer Road, Clifton, Bristol 8. |
| *DIXON, T. B. | 11 Pembroke Road, Clifton, Bristol 8. |
| *DODD, GRANTHAM, M.B., B.S. | 42 Woodstock Road, Bristol 6. |
| *DUERDEN, J. R., M.B., Ch.B. Brist. | 4 Cecil Road, Bristol 8. |
| *DUNLEVY, A. A., M.B., B.Ch. N.U.I. | Ancoats Hospital, Manchester 4. |
| *DUTTON, HILDA R. | 405 Stapleton Road, Bristol 5. |
| *DYER, W. P. | West View, Westbury-on-Trym, Bristol. |
| *EGLINTON, J. H. C. | Street, Somerset. |
| ELLIOTT, F. P., M.B., C.M. Aberd. | 113 Grove Road, Walthamstow, London, E. 17. |
| *ELLIOTT, W. H. A., M.B., B.S. Lond. | 116 Pembroke Road, Clifton, Bristol 8. |
| *ELLIS, W. T. | Tregenna House, Shirehampton. |
| *EVANS, G. M., M.B., Ch.B. Brist. | 117 Ashley Road, Bristol. |
| *EYRE-BROOK, A. L., M.B., M.S. Lond. | The Cottage, Portishead. |
| *FALLON, Col. J. | 35 Henleaze Gardens, Henleaze, Bristol. |
| *FAULL, J. L. | 61 Cotham Brow, Bristol 6. |

| | |
|---|---|
| *FAWN, G. F. .. .. .. .. .. .. .. .. .. .. | 6 Oakfield Road, Clifton, Bristol 8. |
| *FELLS, G. R., M.B., Ch.B. Brist. .. .. .. | 19 Southmead Road, Bristol. |
| *FELLS, R. R., M.B., B.S. Lond. .. .. .. | 17 Mortimer Road, Clifton, Bristol 8. |
| *FENELEY, G. L., M.B., Ch.B. Brist. .. .. | Englewood, Falcondale Road, Westbury-on-Trym. |
| *FINZEL, H. F. M., M.D. Brist. .. .. .. .. .. | Nare House, Birchwood Road, St. Anne's Park, Bristol 4. |
| *FIRTH, J. L., M.D., M.S. Lond. .. .. .. | 8 Victoria Square, Clifton, Bristol 8. |
| *FISHER, A. C., M.D. Brist. .. .. .. .. .. | Roan Antelope Mine, N. Rhodesia. |
| *FISHER, J. A., M.D., B.Sc. Brist. .. .. .. | Canynge Hall, Bristol 8. |
| *FITZGIBBON, G. M., M.D. Lond. .. .. .. | 75 Pembroke Road, Clifton, Bristol 8. |
| *FITZGIBBON, L. PRISCILLA, M.B., B.S. Lond. .. | 75 Pembroke Road, Clifton, Bristol 8. |
| *FORRESTER-BROWN, MAUD, M.D., M.S. Lond. | 22 Combe Park, Bath. |
| *FORSTER, DAIREEN .. .. .. .. .. .. .. .. | Northam House, Henleaze Road, Bristol. |
| *FOSS, G. L., M.B., B.Ch. Cantab. .. .. .. | Cloud's Hill House, St. George, Bristol 5. |
| *FOX, F. E., B.A. Cantab. .. .. .. .. .. .. | Brislington House, Brislington, Bristol 4. |
| *FRASER, A. D., M.A., M.B., Ch.B. Glasg. .. | 4 Alexandra Road, Clifton, Bristol 8. |
| *FRASER, D., M.B., Ch.B. Edin. .. .. .. | Westgate Dursley, Glos. |
| *FULLER, H. L. .. .. .. .. .. .. .. .. .. .. | 19 The Circus, Bath. |
| *FURNIVAL, Col. C. H. .. .. .. .. .. .. .. | "Yatton," Wellington Hill Horfield, Bristol 7. |
| | |
| *GARDEN, R. R., M.A., M.B., B.Ch. Aberd. .. | 9 Harley Place, Clifton, Bristol 8. |
| GARLAND, E. B., M.B., C.M. Edin. .. .. .. | 114a Hampton Road, Redland, Bristol 6. |
| *GERRISH, NORMAN, M.B., Ch.B. Brist. .. .. | 2 Station Road, Keynsham, Som. |
| *GIBB, HELEN M., M.B., Ch.B. Glasgow .. .. | 10 Hill View, Henleaze, Bristol. |
| *GIBBS, J. R. .. .. .. .. .. .. .. .. .. .. | 48 Codrington Road, Bishopston, Bristol 7. |
| *GORDON, R. G., M.D., B.Sc. Edin. .. .. .. | 2 Catharine Place, Bath. |
| *GORHAM, A. P., M.B., Ch.B. Brist. .. .. .. | 53 Westbury Road, Westbury-on-Trym. |
| *GORNALL, W. A., M.D. Brist. .. .. .. .. .. | Ulvik, Nailsea, near Bristol. |
| *GREEN, S. B., M.B. Lond. .. .. .. .. .. .. | The Black Wicket, Westbury-on-Trym. |
| *GREENLAW, MADGE E., M.B., Ch.B. Brist. .. | 206 Redland Road, Bristol 6. |
| GRIFFITHS, CORNELIUS A. .. .. .. .. .. .. | 35 Newport Road, Cardiff. |
| *GROVES, E. W. HEY, M.D., M.S., D.Sc. Lond. .. | 25 Victoria Square, Clifton, Bristol 8. |
| | |
| *HALL, E. GEORGE, M.B. Lond. .. .. .. .. .. | 42 Apsley Road, Clifton, Bristol 8. |
| *HAM, C. I. M.B., Ch.B. Brist. .. .. .. .. .. | Frenchay Park Sanatorium, Frenchay, near Bristol. |
| *HARRIS, H. ELWIN, M.A., M.B. Cantab. .. | The Mount, Halse, Taunton. |
| *HARRIS, H. E., M.A., M.B., B.Ch. Cantab. .. | 13 Lansdown Place, Clifton, Bristol 8. |
| *HARTLEY, GRACE, M.B., B.S. Lond. .. .. | 49 Queen's Court, Clifton, Bristol 8. |
| *HEALES, J. N. .. .. .. .. .. .. .. .. .. .. | Holmcroft, Street, Somerset. |
| *HECTOR, F., M.D. Lond. .. .. .. .. .. .. .. | 1 Victoria Square, Clifton, Bristol 8. |
| *HEMPHILL, R., M.D. Dub. .. .. .. .. .. | Molitor, Barrow Gurney, near Bristol. |
| *HENDERSON, JAMES, M.B., Ch.B. Aberd. .. | The City Sanatorium, Yardley Road, Birmingham. |
| *HERAPATH, C. E. K., *M.C.*, M.D. Lond. .. | Ormlie, Clifton, Bristol 8. |
| *HERON, GORDON, M.B., Ch.B. Brist. .. .. | 26 Henbury Road, Westbury-on-Trym, Bristol. |
| *HERON, DORIS, M.B., Ch.B. Brist. .. .. .. | 26 Henbury Road, Westbury-on-Trym, Bristol. |
| *HEWER, Professor T. F., M.D. Brist. .. .. | Canynge Hall, Bristol 8. |
| *HILL, WINIFRED, M.B., Ch.B. Brist. .. .. | c/o Lloyd's Bank, Ilford. |
| *HOFFMAN, H. LOVELL, B.A., M.B., B.Ch. Cantab. | 26 The Circus, Bath. |
| *HOLLAND, W. A. L. .. .. .. .. .. .. .. .. | 10 Brunswick Square, Bristol. |
| *HOOKER, J. A., M.B., Ch.B. Brist. .. .. .. | 16 St. Edyth's Road, Sea Mills 9. |
| *HOSKEN, J. G. F... .. .. .. .. .. .. .. .. | Uley, Glos. |
| HOSPITAL, BRISTOL GENERAL (*Secretary*, T. W. GREGG). | |
| *HOUGHTON, LYDIA M., M.B., B.S. Lond. .. | 74 Church Road, Redfield, Bristol 5. |
| *HOWARD, C. R. GRENVILLE, M.B. .. .. .. .. | 8 Mortimer Road, Clifton, Bristol 8. |
| *HOWELL, H. .. .. .. .. .. .. .. .. .. .. .. | Kincraig, Beach Road, Weston-super-Mare. |
| *HUGHES, F. W. TERRELL, M.B., Ch.B. Brist. | Chew Magna, Somerset. |
| *HUGHES, MARGUERITE G., M.B., Ch.B. Brist. | 12 Upper Belgrave Road, Clifton, Bristol 8. |

*HUNTER, F. S. .. .. .. .. .. .. .. .. c/o Messrs. J. Wright & Sons Ltd., Publishers, Bristol 1.
*HYATT, ANNIE W., M.B., B.S. Lond. .. .. Merrymead, Shepton Mallet, Som.

*ILES, ANNA, M.B., Ch.B. (Brist.) .. .. .. 87 Pembroke Road, Clifton, Bristol 8.
*ILES, A. E., *O.B.E.*, M.B., B.S. Lond. .. .. 87 Pembroke Road, Clifton, Bristol 8.
INFIRMARY, BRISTOL ROYAL (*Secretary*, E. C. SMITH).
INGLE, C. DURBAN .. .. .. .. .. .. .. Somerton, Somerset.
*ISSERLIN, B. .. .. .. .. .. .. .. .. .. .. 2 College Road, Bristol 8.

*JACKMAN, W. A., M.B., Ch.B. Brist. .. .. 15 Mortimer Road, Clifton, Bristol 8.
*JAMES, APRIL D., M.B., Ch.B. Brist. .. .. (Address unknown.)
*JAMES, J. A., M.D. Lond... .. .. .. .. Litfield House, Clifton Down, Bristol 8.
*JAMES, J. A., Senr. .. .. .. .. .. .. .. .. 43 Nevil Road, Bishopston, Bristol.
*JENKINS, F. G., M.B., Ch.B. Brist. .. .. .. 51 Redcliff Hill, Bristol 1.
*JENKINS, R. D. .. .. .. .. .. .. .. .. .. The Copper Beeches, Almondsbury, near Bristol.
*JENKINS, P. K., M.B., Ch.B. .. .. .. .. 29 Downs Park West, Bristol 6.
*JOHNSON, R. G., M.B. Lond. .. .. .. .. 119 Redland Road, Bristol 6.
*JOLL, C. A., M.D., M.S. Lond. .. .. .. .. 64 Harley Street, London, W.1.
*JONES, MEGAN E. .. .. .. .. .. .. .. Homœopathic Hospital, Bristol 2.
*JORDAN, L. R., M.B., Ch.B. Brist. .. .. .. National Temperance Hospital, Hampstead Road, N.W.1.
*JOSCELYNE, P. C., M.B., Ch.B. Brist. .. .. 17 Falcondale Road, Westbury-on-Trym.

*KEMM, N. F., M.B., B.S. Lond. .. .. .. 200 Redland Road, Durdham Park, Bristol, 6.
*KERSLEY, G. D., M.D. .. .. .. .. .. .. 6 The Circus, Bath.
KING, E. F. .. .. .. .. .. .. .. .. .. .. .. 2 Upper Wimpole Street, London, W.1.
*KINGSTON, S. H., M.B., Ch.B. Brist. .. .. 3 Whiteladies Road, Clifton, Bristol 8.
*KYLE, H. G., M.A., M.D., B.Ch. Oxon. .. .. 31 Westbury Road, Bristol.

*LACE, F .. .. .. .. .. .. .. .. .. .. .. 5 Gay Street, Bath.
LALONDE, T. P., M.B., Ch.B. Brist... .. .. Wykeham House, Romsey, Hants.
*LEES, E. LEONARD, M.D., C.M. Edin. .. .. 28 Downs Park West, Bristol 6.
*LEGAT, R. EDDOWES, M.B., C.M. Edin. .. .. 1 Cambridge Mansions. Woodland Road, Bristol 6.
*LEIGHTON, S. T. P., O.B.E., M.B., Ch.B. (Vict.) Lamport Court, Stinchcombe, Glos.
*LEWIS, F. J., M.B., Ch.B. Brist. .. .. .. Canynge Hall, Bristol 8.
*LING, T. M., M.A., B.M., B.Ch. .. .. .. 50 Pembroke Road, Bristol 8.
*LINTON, MARION S., M.B. Lond. .. .. .. 1 Brecon Road, Henleaze, Bristol.
*LISTER, A. E. J., M.B., B.S. Lond. .. .. 86 Pembroke Road, Clifton, Bristol 8.
*LISTER, L. MARGARET .. .. .. .. .. .. 12 All Saints Road, Clifton, Bristol 8.
*LOGAN, H. B. .. .. .. .. .. .. .. .. .. .. Elmwood, Aberdeen Road, Cotham, Bristol 6.
*LOXTON, S. D., M.B., Ch.B. .. .. .. .. .. 17 Oakfield Grove, Clifton. Bristol 8.
*LUCAS, J. E... .. .. .. .. .. .. .. .. .. 48 Coronation Road, Bristol 3.
*LUCAS, J. J. S., M.D. Lond. .. .. .. .. 186 Wells Road, Knowle, Bristol 4.
*LUCAS, J. V... .. .. .. .. .. .. .. .. .. 186 Wells Road, Knowle, Bristol 4.
*LUCAS R. P., M.B., Ch.B. Brist. .. .. .. 15 Edgecumbe Road, Redland Bristol 6.

*MCCREA, PHILLIP, M.D., B.Ch. Dub. .. .. 56 Kellaway Avenue, Bristol 6.
*MCKENZIE, W. G., *M.C.* .. .. .. .. .. .. 10 Woodland Road, Surbiton.
*MACLACHLAN, A. M., M.B., Ch.B. Glasg. .. 65 Redcliff Hill, Bristol 1.
*MCLANNAHAN, J. G. .. .. .. .. .. .. .. The Mount, Stonehouse.
*MACLAREN, CATHERINE J., M.B., Ch.B. (Edin.) Hereford House, Clifton Down.
MACLEAN, SIR EWEN J., M.D., C.M. Edin. .. 12 Park Place, Cardiff.
*MACLEOD, G., M.A. Glas., M.D., M.B. .. .. Wargrave, Clevedon, Somerset.
*MARSHALL, A. H., M.B., Ch.B. Brist. .. .. Odelos, Canford Lane, Bristol.
MARSHALL, A. L., M.B., B.A. Cantab. .. .. Laxfield, Woodbridge, Suffolk.
*MARTIN, J. E., M.B., Ch.B. .. .. .. .. .. 32 Whiteladies Road, Clifton.
*MARTIN, J. J. B., M.A., M.D. Edin... .. .. Bristol Mental Hospital.
*MARTIN, P. S., *M.C.* .. .. .. .. .. .. .. Victoria Quadrant, Weston-super-Mare.
*MATHESON, N. R., M.B., Ch.B... .. .. .. Southmead Hospital, Bristol.
*MEADOWS, G. .. .. .. .. .. .. .. .. .. W. D. & H. O. Wills, No. 1 Factory, East Street, Bedminster, Bristol 3.
MIALL, C. L'O .. .. .. .. .. .. .. .. .. Elm Hayes, Paulton, near Bristol.

| | |
|---|---|
| *MILLING, T., M.B., Ch.B. Belf. .. .. .. .. | 1 Vicarage Road, Southville, Bristol 3. |
| *MOORE, C. A., M.B., M.S. Lond. .. .. .. | South Lodge, Kingsweston Road, Henbury Bristol. |
| *MOORE, L. A., M.B., Ch.B. Brist. .. .. .. | Hillsborough, Cotham Park, Bristol 6. |
| *MOORE, R. H., M.B., Ch.B. Brist. .. .. .. | White Cross Court, Wells Road, Bristol 4. |
| *MORGANS, C. C., M.B., Ch.B. Cantab. .. .. | 65 Lower Redland Road, Bristol 6. |
| *MORRIS, L. N., M.B., Ch.B. Brist. .. .. .. | 13 Belgrave Road, Clifton, Bristol 8. |
| *MUNDY, G. S., M.B., B.Ch. Brist. .. .. .. | Homewood, Coombe Dingle, Bristol. |
| *MYLES, Q. St. L., M.A., B.M., B.Ch. Oxon. .. | 7 Apsley Road, Clifton, Bristol 8. |
| *NEWLANDS, H. J. .. .. .. .. .. .. .. | Moray House, Fishponds, Bristol. |
| *NICHOLSON-LAILEY, J. R. .. .. .. .. .. .. | Elmfield House, South Road, Taunton. |
| *NIXON, DOREEN, M.B., B.S. Lond. .. .. .. | 7 Lansdown Place, Clifton, Bristol 8. |
| *NIXON, J. A., *C.M.G.*, M.D. Cantab. .. .. | 7 Lansdown Place, Clifton, Bristol 8. |
| *NOBLE, J. I., M.B., Ch.B. Liverp. .. .. .. | 102 Pembroke Road, Clifton, Bristol 8. |
| *NORMAN, R. M., M.D. Brist. .. .. .. .. | Highwood, Stapleton Park, Bristol. |
| *NOTT, WINIFRED, M.B., Ch.B. Brist. .. .. | Fassiefern, 8 Rylestone Grove, Stoke Bishop, Bristol 9. |
| *O'DONOHOE, MONICA A., M.B., Ch.B. .. .. | 1 Beaconsfield Road, Bristol 8. |
| *ORMEROD, G. L. M.A., M.B., B.Ch. Cantab .. | 2 Henleaze Road, Westbury-on-Trym, Bristol. |
| *ORR-EWING, H. J., *M.C.*, M.D. Lond. .. .. | 1 Beaufort Buildings, Clifton, Bristol 8. |
| *PALIN, A. G., B.M., B.Ch. Oxon. .. .. .. | Litfield House, Clifton Down, Bristol 8. |
| *PARKES, J. A., M.B., Ch.B. Liverp. .. .. | 8 South Road, Redland, Bristol 6. |
| PARKINSON, Lt.-Col. G. S., *D.S.O.*, R.A.M.C. | London School of Hygiene and Tropical Medicine, Keppel Street, London, W.C. 1. |
| *PARRY, R. H., M.B., B.S. Lond. .. .. .. | Public Health Offices, 40 Prince Street, Bristol 1. |
| PARSONS, Sir J. H., M.B., B.S., B.Sc. Lond... | 54 Queen Anne Street, London, W. |
| *PAUL, R. G. .. .. .. .. .. .. .. .. | 23 Pembroke Road, Bristol 8. |
| *PAULI, C. H. .. .. .. .. .. .. .. .. | Turville, Pilning. |
| *PERRY, B., M.D., Ch.B. Brist. .. .. .. .. | 4 Richmond Park Road, Clifton, Bristol 8. |
| *PHILLIPS, L. B., M.B., Ch.B. Brist... .. .. | Holdenhurst, Filton, Bristol. |
| *PHILLIPS, P., M.B., Ch.B. Brist. .. .. .. | Southmead Hospital, Bristol. |
| *PIRRIE, A. L., M.B., Ch.B. Glasg. .. .. | 528 Kensington Hill, Bristol. |
| *POCOCK, J. A., M.A., M.B., B.Ch. Cantab. .. | General Hospital, Bristol. |
| *POLLARD, J., M.B., B.Ch., B.A.O., N.U.I .. | 88 Coronation Road, Bristol 3. |
| PORTER, C. P. .. .. .. .. .. .. .. | 28 Mill Street, Kidderminster. |
| *POTTER, MABEL F., M.B., Ch.B. Brist. .. .. | 79 Pembroke Road, Clifton, Bristol 8. |
| *POWELL, H. F., M.D. Brux. .. .. .. .. | Ellenborough Park, Weston-super-Mare. |
| *PRICE, C. H. G., M.B., Ch.B. Brist. .. .. | 6 Lansdown Place, Bristol 8. |
| *PRICE, N. L., M.B., Ch.B. Brist. .. .. .. | Litfield House, Clifton Down, Bristol 8. |
| *PRIDIE, K. H., M.B., B.S. Lond. .. .. .. | 58 St. John's Road, Clifton, Bristol 8. |
| *PROWSE, D. C., M.B., Ch.B. Brist. .. .. .. | Wigmore House, Thornbury, near Bristol. |
| *PYKE, H. D., M.B., Ch.B. Brist. .. .. .. | 88 Redland Road, Bristol 6. |
| *RANKIN, P. C., M.B., Ch.B. Glasg. .. .. .. | Lawrence Hill House, Bristol. |
| *RAYNER, D. C., Ch.M. Brist. .. .. .. .. | 9 Lansdown Place, Clifton, Bristol 8. |
| *REDMAN, ETHEL, M.B., Ch.B. .. .. .. .. | 55 Bridgwater Road, Bedminster Down, Bristol 3. |
| *REES, W. G., M.B., Lond... .. .. .. .. | Northam House, Henleaze Road, Bristol. |
| *ROBERTS, J. A. F., M.A. Cantab., M.B., Ch.B., D.Sc. Edin. | Stoke Park Colony, Bristol. |
| *ROBERTSON, D., M.B., Ch.B. Edin. .. .. .. | 2 Knowle Road, Knowle, Bristol 4. |
| *ROBERTSON, L. G., M.B., Ch.B. .. .. .. | 162 Redland Road, Bristol 6. |
| *ROGERS, H., M.D. Brist. .. .. .. .. .. | 91 Old Park Ridings, Grange Park, N.21. |
| *ROLFE, R., B.A., M.B., B.C. Cantab. .. .. | Park House, St. Andrew's Road, Avonmouth. |
| *ROWLANDS, R. D. .. .. .. .. .. .. | Elmfield House, South Road, Taunton, Som. |
| *ROWLEY, A. T. .. .. .. .. .. .. .. | 13 Walliscote Road, Weston-super-Mare. |
| *RUDOLF, Capt. G. DE M., R.A.M.C. .. .. .. | No. 2 General Hospital, B.E.F. |
| *RYAN, P. B., M.B., Ch.B. .. .. .. .. .. | Southmead Hospital, Bristol. |
| *SAMPSON, O. E. L. .. .. .. .. .. .. | 77 Stackpool Road, Bristol 3. |
| *SCARFF, G., M.B., Ch.B. Edin. .. .. .. .. | 83 Pembroke Road, Clifton, Bristol 8. |

| | |
|---|---|
| *Shepherd, H. L., M.B., Ch.M. Brist. | 79 Pembroke Road, Clifton, Bristol 8. |
| *Short, A. R., B.Sc., M.D., B.S. Lond. | 69 Pembroke Road, Clifton, Bristol 8. |
| *Smith, T. W., M.D. Lond. | 26 The Circus, Bath. |
| *Smythe, H. J. D., *M.C.*, M.D., M.S. Lond. | 15 Eaton Crescent, Clifton, Bristol 8. |
| *Snawdon, J. W. E., M.B., Ch.B., B.D.S. | 170 Redland Road, Bristol 6. |
| *Soltau, H. K. V., M.D. Lond. | 19 The Avenue, Clifton, Bristol 8. |
| *Somers, Mary A. E. | 2 Ashcombe Road, Weston-super-Mare. |
| *Spoor, A., M.B., B.Ch. Cantab. | The Hydro, Bristol 1. |
| *Statham, R. S. S., *O.B.E.* M.D., M.Ch. Brist. | Cheddar, Som. |
| *Steven, G. D., M.B., Ch.B. Edin., D.P.H. | 6 Upper Church Street, Bath. |
| *Stephens, R. J. | 92 Redland Road, Bristol 6. |
| Stone, Doris M., M.D., B.S. Lond. | Beechwood, Church Street, Epsom. |
| *Strover, H. W. M., *O.B.E.*, M.B., Ch.B. Aberd. | 90 Redland Road, Bristol 6. |
| *Struthers, A. J., M.B., Ch.B. Glasg. | 100 Church Road, Redfield, Bristol 5. |
| *Struthers, Geo., M.B., Ch.B. (Glasgow) | 100 Church Road, Bristol 5. |
| *Sutton, G. E. F., M.D. Lond. | 1 Pembroke Road, Clifton, Bristol 8. |
| *Symes, J. O., M.D. Lond. | 6 Pembroke Vale, Clifton, Bristol 8. |
| *Tallack, R. J. K., M.B., Ch.B. | 9 Christchurch Road, Clifton, Bristol 8. |
| *Tasker, D. G. C., M.B., M.S. Lond. | 9 College Fields, Clifton, Bristol 8. |
| *Taylor, A. L., M.D. Lond. | The General Hospital, Bristol 1. |
| Taylor, A. L. | The Royal Infirmary, Bristol 2. |
| Thin, J. | 54 and 55 South Bridge, Edinburgh. |
| *Todd, A. T., *O.B.E.*, M.D. Edin. | Royal Park House, Clifton, Bristol 8. |
| Trinick, Richard H. | 42 Cherry Valley Gardens, Belfast. |
| Tripp, Dorothy M. H. | Mission Hospital, Karim Nagar, Nizam's Dominions, South India. |
| *Trist, J. R. R., *M.C.* | 120 Henleaze Road, Henleaze. |
| *Tryon, Victoria S., M.B., Ch.B. Brist. | 3 Harley Place, Clifton, Bristol 8. |
| *Walker, Cyril H., B.A., M.B. Cantab. | Harewood, Clapton-in-Gordano, Som. |
| *Walters, C. F. | Downover, Walton Down, near Clevedon. |
| *Wansbrough, T. B., M.B., Ch.B. Brist. | 8 Mortimer Road, Clifton, Bristol 8. |
| Wake, S. C., M.B., Ch.B. Brist. | "Stanhope," Bideford, N. Devon. |
| *Ward, H. W. | Chipping Manor, Wotton-under-Edge. |
| *Warren, R., M.A., M.D. Oxon. | Heathgates, Weston-super-Mare. |
| *Watson-Williams, E., *M.C.*, B.A., M.B., Ch.M. Cantab. | 65 Pembroke Road, Clifton, Bristol 8. |
| Watts, J. B. V. | Frantzina's Rust, Barberton, Transvaal, S. Africa. |
| Weddell, C. W. | Boydville, 46 Miller Street, West Melbourne, Australia. |
| *Wigoder, Sylvia, M.A., M.D., Ph.D. | General Hospital, Bristol 1. |
| *Wilbond, R. G. | 76 Chesterfield Road, St. Andrew's Pk., Bristol. |
| *Williams, I., M.B., Ch.B. Brist. | "Ardrem," Hill View, Henleaze, Bristol. |
| *Williams, S. R., M.B. Lond. | Buckfastleigh, Devon. |
| *Willway, F. W., M.D., M.S. Lond. | Bristol Royal Infirmary. |
| *Wood, D. | 105 Pembroke Road, Clifton, Bristol 8. |
| *Wood, Ursula, M.B., Ch.B. | Henley Lodge, Yatton, Som. |
| *Wood, W. V., *M.C.*, B.A. Cantab. | Henley Lodge, Yatton, Som. |
| *Woodman, Dorothy, M.D. Lond. | Kynance, Downs Cote Park, Westbury-on-Trym, Bristol. |
| *Woolley, A. W., M.B., Ch.B. Brist. | Melbourne House, Wells, Som. |
| *Woolley, W., M.B., Ch.B. Brist. | 239 Cranbrook Road, Redland. |
| *Worthington, Lieut.-Col. F., M.B., Ch.B., Cantab. | Greytrees, Briercliffe Road, Coombe Dingle, Bristol 9. |
| *Wright, A. J., M.B., B.S. Lond. | 2 Clifton Park, Clifton, Bristol 8. |
| *Wright, Winifred M., M.B., B.S. Lond. | Merrymead, Shepton Mallet, Som. |
| *Zealand, L., M.D. Man. | Brecon Lodge, Westbury-on-Trym, Bristol. |

